# Spatial incongruence in the species richness and functional diversity of cricetid rodents

**DOI:** 10.1371/journal.pone.0217154

**Published:** 2019-06-07

**Authors:** Cintia Natalia Martín-Regalado, Miguel Briones-Salas, Mario C. Lavariega, Claudia E. Moreno

**Affiliations:** 1 Centro de Investigaciones Biológicas, Instituto de Ciencias Básicas e Ingeniería, Universidad Autónoma del Estado de Hidalgo, Mineral de la Reforma, Hidalgo, México; 2 Centro Interdisciplinario de Investigación para el Desarrollo Integral Regional, Unidad Oaxaca, Instituto Politécnico Nacional, Santa Cruz Xoxocotlán, Oaxaca, México; University of Sydney, AUSTRALIA

## Abstract

Biodiversity is multidimensional and different mechanisms can influence different dimensions. The spatial distribution of these dimensions can help in conservation decisions through the location of complementary areas with high diversity. We analyzed congruence in spatial patterns of species richness and functional diversity of cricetid rodents in the state of Oaxaca, southern Mexico, at different scales, and environmental variables related. Potential distribution models were produced for 49 species of cricetids in Maxent and superimposed to obtain potential communities in cells of 25, 50,100, 200 and 400 km^2^. We estimated species richness (SR) and functional diversity (SES.FD) eliminating the species richness effect through null models. The patterns and spatial congruence of species richness and functional diversity are described. The relationships between the environmental variables (elevation, temperature, precipitation, net primary productivity and potential evapotranspiration) and the SR and SES.FD were explored using Generalized Linear Models (GLMs) and Generalized Additive Models (GAMs). The highest species richness was found in mountainous ecosystems while the highest functional diversity was in tropical forests, revealing a spatial incongruence among these components of biodiversity (r = -0.14, *p* = 0.42; Pearson correlation). The locations of the cells of low congruence varied according to spatial resolution. In univariate models, elevation was the variable that best explained species richness (R^2^ = 0.77). No single variable explained the functional diversity; however, the models that included multiple environmental variables partially explained both the high and low functional diversity. The different patterns suggest that different historic, ecological and environmental processes could be responsible for the community structure of cricetid rodents in Oaxaca. These results indicate that one great challenge to be met to achieve more effective planning for biological conservation is to integrate knowledge regarding the spatial distribution of different dimensions of biodiversity.

## Introduction

Understanding the processes and mechanisms that generate the spatial patterns of species richness is a central theme in biogeography and macroecology [1–3]. For example, the species-area relationship, biotic and abiotic determinants, and latitudinal gradients in species richness are recurrent and widely studied patterns [4]. However, this approach has been focused only on the number of species. Therefore, to achieve a fuller understanding of the spatial patterns of biodiversity and its determinants, i.e., the environmental factors that regulate biodiversity, the spatial distribution of other facets or dimensions of biodiversity has become the subject of recent studies [5–10]. One of these dimensions is functional diversity, in which species are characterized by their functional traits, that are suspected to be relevant in their performance in specific habitats, providing a greater understanding of the links that exist between biodiversity and ecosystem functioning [8, 11–13].

In mammals, the spatial patterns of species richness have been widely studied [8, 14, 15]. For example, with respect to the altitudinal species diversity gradient in rodents, it has been shown that the highest concentration of species occurs at intermediate altitudes [16–18]. Some studies also provide evidence of the influence of determinant environmental factors on spatial patterns. These factors include climate, productivity and habitat heterogeneity [19–22]. However, it has recently been found that the species richness and functional diversity of mammals do not have a marked spatial congruence. Therefore, a disparity or spatial mismatch is often found in these dimensions of diversity; while species richness often correlates closely with environmental conditions, such as elevation, temperature and productivity, functional diversity depends on both environmental conditions and ecological interactions among coexisting species, such as competence [7, 8, 10, 23].

However, there has been little attention to the spatial patterns of functional diversity in small mammals (e.g., [6]), such as the Family Cricetidae (Rodentia), one of the most diverse in North America [24] with about 310 genera and approximately 1,517 species [25]. Due to their high taxonomic diversity, morphological and evolutionary variation, and wide distribution, cricetid rodents constitute a suitable group for studying patterns of diversity. In Mexico, the highest richness of cricetid rodents occurs in the southern states, of which Oaxaca harbors the greatest specific richness (49 species in 15 genera), followed by Chiapas (35 species) and Veracruz (34 species) [26–30]. Also, Oaxaca has high environmental heterogeneity. Thus, this region is an ideal scenario to explore the functional diversity of cricetid communities and its drivers.

The objectives of this study were: 1) to describe the spatial patterns of the species richness and functional diversity; 2) to evaluate the spatial congruence of the species richness and functional diversity; 3) to evaluate the effect of spatial scale on patterns of species richness and functional diversity in order to assess whether the results are affected by the size of cells; and 4) to evaluate the relationships between elevation, temperature, precipitation, net primary productivity and potential evapotranspiration and species richness and functional diversity. It is expected that the richness of cricetid rodents will be explained by elevation [31–33] while the functional diversity will be determined by a set of environmental factors. In addition, it has been seen that the spatial patterns of richness and functional diversity are unrelated (i.e., are spatially incongruent) in other groups of mammals with lower diversity [10], and therefore we assume that the lack of spatial congruence between these two dimensions of biodiversity will be even higher in a more diverse group, such as the cricetid rodents.

## Materials and methods

### Study area

**Fig 1:**
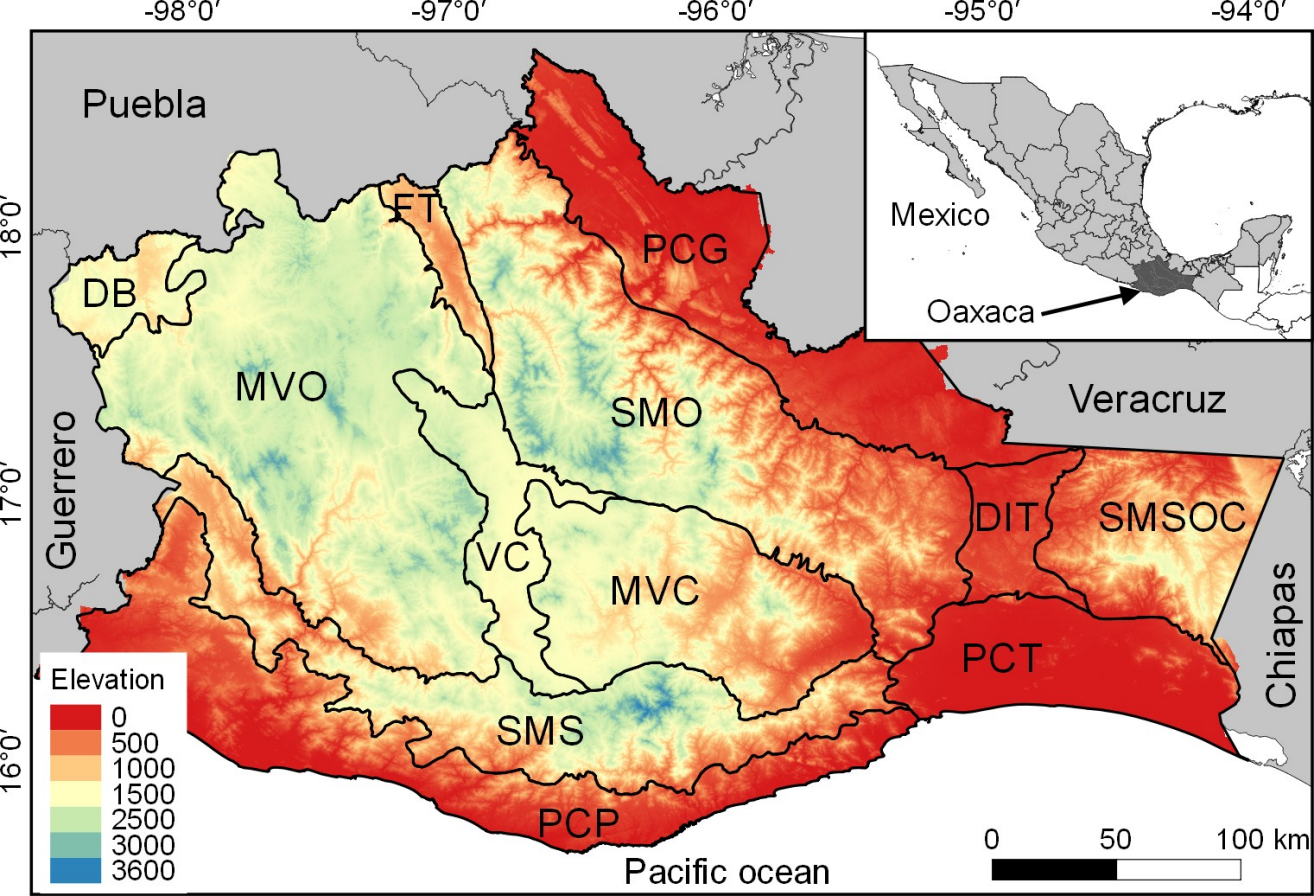
Location of Oaxaca, including the physiographic subprovinces of the state: Depresión del Balsas (DB), Montañas y Valles del Occidente (MVO), Fosa de Tehuacán (FT), Sierra Madre de Oaxaca (SMO), Valles Centrales de Oaxaca (VCO), Montañas y Valles del Centro (MVC), Sierra Madre del Sur (SMS), Planicie Costera del Pacífico (PCP), Planicie Costera de Tehuantepec (PCT), Depresión del Istmo de Tehuantepec (DIT), Sierra Madre del Sur de Oaxaca y Chiapas (SMSOC), and Planicie Costera del Golfo (PCG).

The state of Oaxaca is located in southern Mexico, between the geographic coordinates 15°39’and 18°39’ N and 93°52’and 98°32’ W. It has an area of 95,364 km^2^, which represents 4.8% of the national territory [34]. The topography is heterogeneous, with elevations ranging from sea level up to 3,600 m a.s.l. (Fig 1). The state presents 26 climate types, from warm and dry on the Pacific coastal plain to cold and humid on the mountain tops. Due to its complex orography, the territory has been divided into 12 physiographic subprovinces that are distinguished by their particular geomorphological traits [35]. For more details, see S1 Table.

### Databases

Species occurrence records were obtained for 49 species of cricetid rodents in the state of Oaxaca from the mammal collection of the Centro Interdisciplinario de Investigación para el Desarrollo Integral Regional, Unidad Oaxaca (OAX.MA.026.0497, CIIDIR-Oaxaca, IPN; https://www.ciidiroaxaca.ipn.mx/mastozoologia/) and of Global Biodiversity Information Facility (https://www.gbif.org). In the page of OAX.MA, in the section “Representación taxonómica y geográfica”, there is a systematic list of terrestrial mammals deposited in the collection, and to request information about the specimens it is necessary to send an email to the curator (mbriones@ipn.mx; coleccionmamiferos_ciidiroax@ipn.mx). In the page of GBIF, it is necessary to search the species, and then in the section of occurrences it is possible to download the records. As quality control, the records were reviewed taxonomically and geographically, selecting those occurrences that met the following criteria: 1) have been obtained after 1950, 2) with geographical coordinates corresponding to the locality referred, and 3) localities < 15 km in a straight line from the town of reference. In all the cases, the geographical coordinates were reviewed; mistakes were corrected and, when geographical coordinates were lacking, the occurrences were georeferenced. All the occurrences were geographically validated based on collection locations, using vector maps of municipalities, localities, gazetteers, and Google Earth. We extracted the following information from the final databases: locality, coordinates, year, and scientific name.

### Species distribution modeling

Functional diversity studies have been based on expert-based maps (IUCN Red List Threatened Species); these maps are adequate for coarse-scale macroecological studies (1-degree resolution), but at finer resolutions these maps may give imprecise spatial patterns [36, 37]. Therefore, we used species distribution modeling to construct species range maps with a finer resolution, appropriate to the objective of this study.

The species’ potential geographic distribution (areas that have environmental conditions very similar to the sites where the species are found [38, 39] of the 49 species of cricetids were estimated with Maxent 3.3.3 software [40]. Maxent has a good performance to predict the species potential distribution, even with small sample sizes [40–42]. Nineteen bioclimatic variables and elevation were obtained from WorldClim (version 1; http://www.worldclim.org/). The bioclimatic variables were built with climatic data gathered between 1950 and 2000, and all of them have a spatial resolution ~1 km^2^ [43]. The modeling area delimitation was species-specific, taking into consideration the biogeographic history of each species [44]. This modeling area (or accessible area sensu [44]) corresponds to the geographical zones where the species is, or is supposed to be, given their dispersion capacities and the absence of large environmental barriers or discontinuities that could limit their establishment in geological times [45]. In our paper, we used the American physiographic provinces of [46]. The environmental variables were cut with the accessible area in QGIS [47]. Subsequently, for these variables, a correlation analysis was performed in the ENMTools software [48], and when a pair of variables showed high correlation (r≥0.90), only the variable with most biological significance for the species was selected. This procedure prevented errors in the predictions due to overfitting of the models [49]. On the other hand, the validated occurrences were overlaid on the environmental variables and the values were extracted using the Point Sampling Tool in QGIS. With this, environmentally correlated records were identified and discarded.

In Maxent, different percentages of records for training were tested (60, 70, 75 and 80%), having as parameters the automatic characteristics, logistic output format, ASCII output file format, one regularization, one replicate, and 10,000 maximum points of background. The evaluation of the models was done with the Area Under the Curve (AUC) of the Receiver Operating Characteristic (ROC) [40]. The models with the highest AUC (>0.90) and showing best qualitative fitting to expert maps [50–52] were reclassified to binary (presence/absence) maps taking as threshold the value of the 10 percentile of the training data for species with > 60 occurrences and with the minimum value of the training data when the species had < 60 occurrences (see S2 Table). Subsequently, in QGIS, the models were transformed to shapefiles and the areas overestimated (where the species could not be accessed due to the presence of geographical barriers) were discarded.

### Mapping

**Fig 2:**
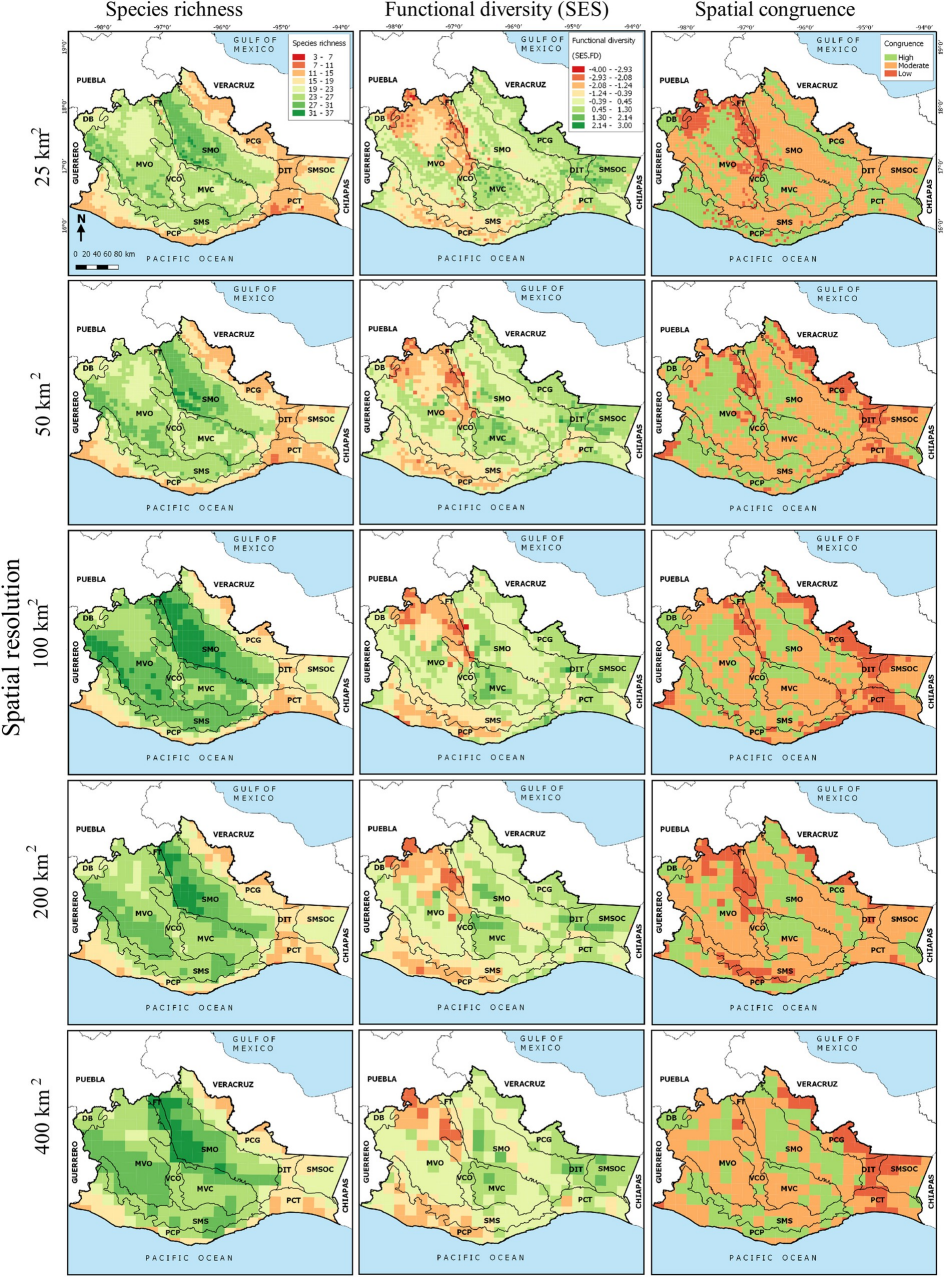
Species richness, functional diversity and spatial congruence of cricetid rodents communities in the state of Oaxaca, México, at different spatial resolution. The physiographic subprovinces of the state are also delimited: Depresión del Balsas (DB), Montañas y Valles del Occidente (MVO), Fosa de Tehuacán (FT), Sierra Madre de Oaxaca (SMO), Valles Centrales de Oaxaca (VCO), Montañas y Valles del Centro (MVC), Sierra Madre del Sur (SMS), Planicie Costera del Pacífico (PCP), Planicie Costera de Tehuantepec (PCT), Depresión del Istmo de Tehuantepec (DIT), Sierra Madre del Sur de Oaxaca y Chiapas (SMSOC), and Planicie Costera del Golfo (PCG).

The Figs 1 and 2 were made using QGIS (version 2.18.7; https://qgis.org/es/site/), a free GIS software. Sources of Fig 1 are: 1) a digital elevation model (DEM) map, generated with ASTER images downloaded from https://earthexplorer.usgs.gov; 2) a hill shaded map, generated by us with the aforementioned DEM, and QGIS algorithm; 3) Mexican political limits, obtained from http://www.conabio.gob.mx/informacion/gis/; and 4) a physiographic subprovinces map, generated by us, based on [35]. Sources used to draw Fig 2 are: 1) maps made by us by overlapping species distribution models; 2) Mexican political limits, obtained from http://www.conabio.gob.mx/informacion/gis/; and 3) a physiographic subprovinces map, generated by us, based on [35]. The ASTER images and Mexican political limits are all of public domain.

### Definition of cricetid communities

Grids were drawn with cells of different resolution (see below) covering the entire state of Oaxaca. Each cell was considered an ecological community in which different cricetid species could potentially coexist. Thus, the species richness per cell (SR) is the sum of all of the species with a potential distribution model that at least partially includes that particular cell.

### Functional trait selection

A functional trait is a measurable property that strongly influences the performance of an organism [53]. For the cricetid rodents, morphological measurements were selected as functional traits (Table 1), given that mammal morphology is related to thermoregulation, interspecific exclusion and the capacity for resource use [54–58], as well as biological traits that represent the requirements of the species [59–60]. Rodent morphological traits are also good indicators of functional ecological aspects such as diet, locomotion and substrate use strategies [61]. The functional traits used were: 1) total length, 2) tail length, 3) hind foot length, 4) ear length, 5) weight, 6) zygomatic breadth, 7) pattern of circadian activity, 8) habitat, 9) diet, and 10) number of offspring per litter (Table 1).

**Table 1:** Traits used to quantify the functional diversity of cricetid rodents of Oaxaca, Mexico.

Traits	Value/category (units)	Variable type
1. Total length	Mean (mm)	Continuous
2. Tail length	Mean (mm)	Continuous
3. Hind foot length	Mean (mm)	Continuous
4. Ear length	Mean (mm)	Continuous
5. Weight	Mean (g)	Continuous
6. Zygomatic breadth	Mean (mm)	Continuous
7. Pattern of circadian activity	Cathemeral	Categorical
	Crepuscular	Categorical
	Diurnal	Categorical
	Diurnal-crepuscular	Categorical
	Nocturnal	Categorical
8. Habitat	Arboreal	Categorical
	Semiaquatic	Categorical
	Semiarboreal	Categorical
	Terrestrial	Categorical
	Terrestrial-semiaquatic	Categorical
	Terrestrial-semiarboreal	Categorical
9. Diet	Carnivore	Categorical
	Frugivore	Categorical
	Frugivore-granivore	Categorical
	Granivore	Categorical
	Herbivore	Categorical
	Herbivore-insectivorous	Categorical
	Insectivorous	Categorical
	Omnivore	Categorical
10. Number of offspring per litter	Mean	Continuous

In order to obtain the values of morphological functional traits, when they were available, 10 adult specimens (5 males and 5 females) selected by random of each species were measured from the mammal collections of the Centro Interdisciplinario de Investigación para el Desarrollo Integral Regional, Unidad Oaxaca (OAX.MA, CIIDIR-Oaxaca, IPN), the Instituto de Biología (CNMA, IBUNAM) and the Museo de Zoología “Alfonso L. Herrera” of the Facultad de Ciencias (MZFC-M), both of the Universidad Nacional Autónoma de México. For the first six traits, the mean values of each measurement were obtained (Table 1). For the other four traits, the information was obtained from published literature [62–66]. In eight species no information was available, and the data of taxonomically closely related species were used [26, 67].

### Calculation of functional diversity and congruence with species richness

The index of functional diversity (FD) of Petchey and Gaston [68, 69] was calculated for each of the communities; this measured the total length of the branches that unite all of the species on a functional dendrogram of the community (a multivariate analysis of classification based on functional traits; [68, 69]). The Gower distance was used, since this is recommended for a combination of quantitative and qualitative data [70–72]. In order to create the distance matrix and construct the dendrogram, the Unweighted Pair Group Method with Arithmetic Mean (UPGMA) clustering procedure was used [72–75]. The FD index is a common metric of functional diversity due to its performance with multiple traits [8, 10, 76–80].

The FD index generally correlates positively with species richness [81]. For this reason, a null model approach [82] was used to eliminate the effect of species richness on the FD and to describe the pattern of functional diversity appropriately. The null model selected species at random but maintaining the species richness for each community [77, 83]. To calculate the standardized effect size (SES) of FD the following formula was used: SES.FD = (Mean_obs_-Mean_null_)/sd_null_, where Mean_obs_ is the mean of observed measurements in a certain species assemblage; Mean_null_ is the mean of 99 iterations generated under the null model; and sd_null_ is the standard deviations of iterations on measures. The results of the null model provide the SES, standard deviation and the p-value for each community. Positive values of SES.FD indicate that the functional diversity is greater than that expected by chance (functional overdispersion), while negative values of SES.FD represent a lower functional diversity than would be expected by chance (functional clustering). The SES.FD is an effective form of comparing the FD of the communities while eliminating the bias associated with differences in richness [83, 84].

In order to evaluate the spatial congruence of species richness (SR) and functional diversity without the effect of the richness (SES.FD), the values of both measurements were classified into three categories of equal intervals: high, moderate and low. It was considered that there is high spatial congruence between SR and FD when the categories for both measures were identical (high SR and high FD, moderate SR and moderate FD, or low SR and low FD); moderate congruence corresponded to categories of SR and FD contiguous (high SR and moderate FD, moderate SR and high FD, moderate SR and low FD, or low SR and moderate FD); finally it was considered as a spatial incongruence when the categories among both measures were extreme (SR high and FD low, and SR low and FD high). The same procedure was used to evaluate the congruence between SR and SES.FD at different spatial resolution (results of the evaluation are shown in S3 Table).

### Evaluation of spatial scale in species richness and functional diversity

Some studies have shown that spatial incongruence can be an artifact of spatial scale [85, 86], i.e., the size of the cells used in the study. If scale were an important factor, we would therefore expect that the use of fine-grained cells would produce spatial incongruence, this because the composition of the communities is more variable, while greater congruence would be found using coarse-grained cells.

In order to test this hypothesis, patterns of species richness and functional diversity in cricetid rodents were compared at different scales, using cells of 25, 50, 100, 200 and 400 km^2^ (i.e., of different spatial resolution). The number of cells in each case was 3,967, 2,051, 1,092, 548 and 283, respectively. Species richness and functional diversity were obtained for each cell, and the spatial congruence was evaluated between both measurements, following the procedures described above. In order to compare maps of different scales, the minimum and maximum ranges were visualized.

### Environmental variables and their relationship with richness and functional diversity

The influence of environmental variables on the cricetid communities was explored only in communities with cells of 100 km^2^, since this spatial resolution covers the geographic range of the microendemic species and this cell size has been used previously in studies with small mammals [7, 8]. Thus, the following environmental variables were obtained for each 100 km^2^ cell: 1) elevation, 2) temperature, 3) precipitation, 4) net primary productivity and 5) potential evapotranspiration (Table 2).

**Table 2:** Environmental variables used to evaluate the relationship between the species richness and the functional diversity in cricetid rodents in Oaxaca, México.

Variable	Description	Resolution	Source
Elevation	The mean elevation value per cell	~1 km	[43]
AMT	Annual mean temperature value averaged per cell	~1 km	[43]
AMP	Annual mean precipitation value averaged per cell	~1 km	[43]
NPP	Net primary productivity	~1 km	https://earthexplorer.usgs.gov
PET	The potential evapotranspiration mean per cell	~0.5 km	https://earthexplorer.usgs.gov

These variables were selected because previous studies with mammals showed that they are significant predictors of functional diversity [8, 10, 60]. To determine the relationship between the measurements of diversity (SR and SES.FD, this last were separated to disentangle which variables are explaining the functional grouping and which ones the functional dispersion, separately) and the environmental variables, Generalized Linear Models (GLMs) and Generalized Additive Models (GAMs) were used. The GAMs are non-parametric extensions of GLMs that are used when there are no linear patterns between the response and explanatory variables, or when these are revealed by examination of the diagnostic graphs of a GLM [87] (see S1 Text). We compared the GLMs and GAMs for each dependent variable according to their Akaike values (AIC). For SR we used a Poisson distribution as this is a discrete probability distribution useful for describing count data, such as the number of species, whereas for SES we used Gaussian distributions as the SES are continuous. Moreover, because we detected overdispersion, we corrected the standard errors using a quasi-model. The geographic coordinates of the centroids of the cells were included as a uniform factor to control spatial autocorrelation [88]. In addition, we performed a correlation corrected with the Tjostheim’s coefficient for the spatial autocorrelation between the species richness and the SES.FD. This correction was necessary because the spatial autocorrelation in both measurements could act to increase the rates of Type 1 statistical error [89].

In an initial phase, the GLMs and GAMs were conducted separately with each environmental variable (univariate models). Subsequently, we did multivariate models. For this case, we tested the collinearity among variables with Pearson correlations, which showed a high correlation coefficient between elevation and temperature (-0.97; see S4 Table). For this reason, elevation was eliminated from multivariate analyses. We tested all possible combinations of the four environmental variables, and the model with the lowest value of AIC was chosen (see S5 Table). All analyses were conducted with the software R using the packages vegan, mgcv and SpatialPack [90].

## Results

We obtained 23,108 records of 49 cricetid rodent species. Of this total, 8,509 records were spatially unique, and of these 3,380 were used to model potential species distributions. *Microtus umbrosus* was the only species for which we did not construct a distribution model because its records were restricted to a single locality, therefore, the distribution area was considered as a single cell (100 km^2^) (Table 3).

**Table 3:** Taxonomic list of cricetid species for the state of Oaxaca (following the nomenclature of Ramírez-Pulido [26]). The records were downloaded from the GBIF and provided by the OAXMA. The numbers of distinct localities are validated records.

Species	Number of records	Number of distinct localities	Records used in modelling
1. *Microtus mexicanus* (de Saussure, 1861)	690	328	98
2. *Microtus oaxacensis* Goodwin, 1966	144	24	11
3. *Microtus quasiater* (Coues, 1874)	530	18	18
4. *Microtus umbrosus* Merriam, 1898	1	1	1
5. *Baiomys musculus* (Merriam, 1892)	925	703	86
6. *Scotinomys teguina* (Alston, 1877)	91	63	29
7. *Hodomys alleni* (Merriam, 1892)	79	68	31
8. *Neotoma mexicana* Baird, 1855	608	312	126
9. *Habromys chinanteco* (Robertson and Musser, 1976)	5	5	5
10. *Habromys ixtlani* (Goodwin, 1964)	301	17	17
11. *Habromys lepturus* (Merriam, 1898)	5	5	5
12. *Habromys simulatus* (Osgood, 1904)	11	7	7
13. *Megadontomys cryophilus* (Musser, 1964)	316	22	20
14. *Megadontomys nelsoni* (Merriam, 1898)	14	10	10
15. *Megadontomys thomasi* (Merriam, 1898)	79	60	54
16. *Peromyscus aztecus* (de Saussure, 1860)	2,033	244	211
17. *Peromyscus beatae* Thomas, 1903	119	83	78
18. *Peromyscus difficilis* (J. A. Allen, 1891)	494	254	94
19. *Peromyscus furvus* J. A. Allen and Chapman, 1897	217	163	155
20. *Peromyscus gratus* Merriam, 1898	514	282	189
21. *Peromyscus leucopus* (Rafinesque, 1818)	1,379	826	56
22. *Peromyscus maniculatus* (Wagner, 1845)	911	579	130
23. *Peromyscus megalops* Merriam, 1898	1,084	92	76
24. *Peromyscus melanocarpus* Osgood, 1904	2,800	61	54
25. *Peromyscus melanophrys* (Coues, 1874)	479	331	104
26. *Peromyscus melanotis* J. A. Allen and Chapman, 1897	409	229	61
27. *Peromyscus melanurus* Osgood, 1909	148	17	17
28. *Peromyscus mexicanus* (de Saussure, 1860)	1,115	518	124
29. *Reithrodontomys fulvescens* J. A. Allen, 1894	1,107	641	204
30. *Reithrodontomys megalotis* (Baird, 1857)	1,098	546	67
31. *Reithrodontomys mexicanus* (de Saussure, 1860)	171	129	100
32. *Reithrodontomys microdon* Merriam, 1901	201	35	35
33. *Reithrodontomys sumichrasti* (de Saussure, 1860)	602	382	42
34. *Oligoryzomys fulvescens* (de Saussure, 1860)	35	26	26
35. *Oryzomys alfaroi* (J. A. Allen, 1891)	318	177	129
36. *Oryzomys chapmani* Thomas, 1898	1,458	97	65
37. *Oryzomys couesi* (Alston, 1877)	876	287	203
38. *Oryzomys guerrerensis* Goldman, 1915	52	21	21
39. *Oryzomys melanotis* Thomas, 1893	234	57	56
40. *Oryzomys fulgens* Thomas, 1893	21	16	16
41. *Oryzomys rostratus* Merriam, 1901	57	43	42
42. *Rheomys mexicanus* Goodwin, 1959	3	3	3
43. *Sigmodon alleni* Bailey, 1902	371	47	47
44. *Sigmodon leucotis* Bailey, 1902	93	44	44
45. *Sigmodon mascotensis* J. A. Allen, 1897	262	161	146
46. *Sigmodon planifrons* Nelson and Goldman, 1933	3	3	3
47. *Sigmodon toltecus* (de Saussure, 1860)	286	198	127
48. *Nyctomys sumichrasti* (de Saussure, 1860)	184	148	83
49. *Tylomys nudicaudus* (Peters, 1866)	176	127	55
Total	23,108	8,509	3,380

### Spatial patterns of species richness and functional diversity

Superposition of the potential distribution models of the 49 species showed greater species richness of cricetid rodents in the mountainous areas, within the physiographic subprovinces Sierra Madre de Oaxaca (a mountainous area with an average elevation above 2,500 m a.s.l.), Montañas y Valles del Occidente (a system of mountains that form a cusp at their point of convergence) and Sierra Madre del Sur (where the relief is contrasting but essentially the mountains have an average altitude of 2,000 m) (Fig 2). The distribution of species richness on the grids was spatially heterogeneous (e.g., using the resolution of 100 km^2^, the mean value was 23 species per cell, minimum 10 and maximum 36 species). The highest values of functional diversity without the effect of richness (SES.FD) were found in the tropical forests of the Montañas y Valles del Centro (with a maximum altitude of 2,800 m a.s.l., with climate that ranges from temperate to warm-dry) and the Sierra Madre del Sur de Oaxaca y Chiapas (altitudes generally below 1,000 m a.s.l.) (Fig 2).

We performed a preliminary analysis of spatial congruence between the SR and the FD using the 100 km^2^ cells (see S1 Fig). This analysis revealed high congruence between both measurements (in 22.95% of the cells with low richness and low FD, and 33.97% of the cells with high richness and high FD), and intermediate congruence in 16.09% of cells, while any of the cells had incongruence (S3 Table) since these two variables are highly correlated (r = 0.99, *p*<0.001). We are not adding to this high congruence numbers those cells with moderate richness and moderate SES.FD because they could randomly inflate these results due to the high chance of overlap in the center of the distribution of values.

**Fig 3:**
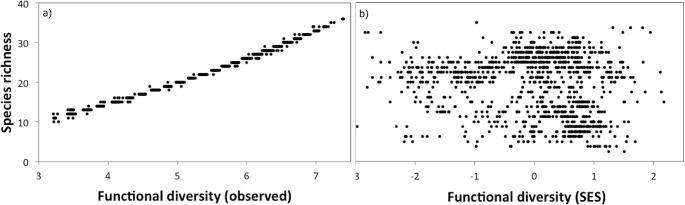
**Dispersion of data by comparing species richness and observed functional diversity (a) and species richness and functional diversity without the effect species richness (b; SES.FD metric)**.

Analysis of spatial congruence was therefore conducted between the SR and functional diversity, but without the effect of richness (SES.FD metric). The pattern obtained was different for the 100 km^2^ cells, with spatial incongruence in 18.32% of the cells, moderate congruence in 51.55% and high congruence in only 1.74% of cells with low richness and low SES.FD, and 10.62% of cells with high richness and high SES.FD (S3 Table), and there was no significant correlation between these variables (r = -0.14, *p* = 0.42; corrected correlation = -0.085). The dispersion of these data is presented in Fig 3.

### Species richness and functional diversity at different scales

The spatial patterns of species richness and functional diversity in cells of 25, 50, 200 and 400 km^2^ were similar to those described for the cells of 100 km^2^ (Fig 2). For this reason, the same physiographic subprovinces that had high species richness and high functional diversity (SES.FD) in cells of 100 km^2^ had high values in both measurements in the different spatial scales analyzed.

Contrary to our expectation, we did not find a clear effect of scale on the spatial congruence between SR and SES.FD: 0.20% of cells with low-low values in richness and SES.FD, and 7.18% of cells with high-high values in these measures at 25 km^2^; 0.20% low-low cells and 14.58% high-high cells in 50 km^2^; 0.73% low-low cells and 12.78% high-high cells for 200 km^2^; and only 9.57% of high-high cells for 400 km^2^. At all scales, at least half of the cells showed moderate congruence, while the percentage of cells with incongruence between SR and SES.FD varied from 6.2 to 16.24% (corresponding to cells of 25 and 200 km^2^, respectively). However, beyond the percentage of cells with congruence, it is important to highlight the effect of scale on the location of these cells. With the fine resolution of 25 km^2^, the cells of low congruence between SR and SES.FD are found in the east of Oaxaca, in the Fosa de Tehuacán (which has elevations below 1,000 m a.s.l., in which a semiarid climate dominates) and in the north of Montañas y Valles del Occidente and of Valles Centrales de Oaxaca (where most of the area is below 1,600 m a.s.l., and the climate is warm-dry). Using cells of intermediate resolution, we found low congruence in the Planicie Costera del Pacífico y del Golfo (a flat area with a warm dry climate), as well as in the zone of the Tehuantepec Isthmus. With the cells of 400 km^2^, we found low congruence in the Tehuantepec Isthmus and in the cells adjacent to the state of Veracruz. For this reason, we conclude that spatial resolution has a prominent effect on the incongruence between the species richness and functional diversity of cricetids (Fig 2).

### Relationship between environmental variables and species richness and functional diversity

In the univariate models, in some cases the best fit (lowest AIC) was obtained in GLMs and in other cases in GAMs. For species richness, all of the environmental variables were highly significant (S5 Table). Of these variables, elevation explained the greatest variance (R^2^ = 0.77). In contrast, for the positive values of SES.FD, all the environmental variables but temperature were significant to explain the high functional diversity, although R^2^ values were very low; for the negative SES.FD all environmental variables were significant, and precipitation (AMP) was the variable that best explained the low functional diversity (R^2^ = 0.35) (S5 Table).

With multiple variables the GAMs had lower AIC values than GLMs. The best model for species richness, SES positive and negative values included all the four environmental variables (AMT+AMP+NPP+PET; R^2^ = 0.78, 0.27 and 0.46 for species richness, SES positive and negative values, respectively; S5 Table).

## Discussion

According to our expectation regarding lack of spatial congruence between species richness and functional diversity in this highly diverse group of mammals in Oaxaca, by using models of their potential distribution we found notably different spatial patterns. These results support the notion found in other studies that the species richness and functional diversity of mammals are spatially disconnected at a variety of scales, e.g., mammals worldwide [7], medium and large mammals in Mexican Transition Zone [10]. However, potential sample biases on species distribution models due to small occurrences datasets could be an issue. The maps of species with few occurrences could have omission or commission mistakes even if Maxent usually performs well with few occurrences and even with species with restricted spatial distribution patterns [91], such as some species in this work (e.g., *Habromys chinanteco*, *Rheomys mexicanus*, *Sigmodon planifrons*).

The spatial incongruences imply that functional diversity was not necessarily high in communities with high species richness, therefore it can be assumed that species-rich communities such as those of the western Sierra Madre de Oaxaca harbor species with relatively similar functional traits (functional redundancy). This is frequently found in communities with high species richness [92], where various species have similar functional traits, for which reason they could potentially perform similar roles in the ecosystems [93]. In our study, some species of the genus *Peromyscus* (13 species for Oaxaca) often share very similar traits (e.g., they are nocturnal, terrestrial and omnivorous) and morphological and behavioral characteristics, which could indicate a certain degree of ecological redundancy. However, a species that is redundant in one community may not be so in another [94]. For example, some communities of the Planicie Costera de Tehuantepec have different genera, and a few number of species in each genus (on average 2.3 species per genus), and fewer similar traits; in contrast sites with many species belonging to few genera, such as the Sierra Madre de Oaxaca (on average 3.4 species per genus), will have trait redundancy (low functional diversity). Then, the species-genera relationship could indirectly explain the functional diversity found. Thus, the similarity in traits between species of cricetid rodents could be explained from the perspective of phylogenetic conservatism of the niche, which is based on the hypothesis that closely related species are more ecologically similar than could be expected as a function of their phylogenetic relationships [95]. We recommend further studies regarding phylogenetic structure and its possible influence on the spatial incongruence of diversity dimensions, as the evolutionary relationships can be informative for understanding the processes of cricetid diversification due to physiological or ecological traits.

When we evaluated the effect of spatial scale on patterns of species richness and functional diversity, we expected differences in spatial congruence among scales, with more incongruence at fine-grained cells. However, we did not find clear trends to support this idea when we looked at the low-low and high-high values of congruence between species richness and functional diversity, although there were changes in the geographical location of incongruent cells. This could mainly be due to changes in the taxonomic composition of communities at the different scales analyzed. For this reason, the structure of the communities is very important, since this could have implications for the inter- and intraspecific relationships of cricetids in the ecosystems.

The problem of selecting a proper scale could be solved by considering the biology (e.g., microhabitat selection, climate tolerance) and range of the cricetid species, because at coarse scales, cells are more environmentally heterogeneous and variable than at fine scales, then species coexistence is less likely to occur at coarse scales. In this sense, and in terms of the conservation of cricetid rodents in Oaxaca, it is suggested that the best scale is 100 km^2^ because these cells are large enough to include the range of microendemic species and small enough to apply realistic local conservation actions [96]; however, in order to obtain the optimum panorama, it is necessary to employ other approaches at different scales.

Spatial incongruence between species richness and functional diversity is often attributed to ecological mechanisms and/or historic events [97]. To date, it has been found that elevation, precipitation, temperature and evapotranspiration are important determinants of the variation of species richness and functional diversity of mammals [8, 33, 98, 99]. In this study precipitation was positively correlated with FD. Low FD values occurred most in subprovinces with low precipitation in seasonal climates, whereas moderate and high FD values were in subprovinces with high and moderate precipitation levels. The sites with low FD could be regions where species (related or no related) with similar traits are prone to compete, and in the long term, only one species prevailed, thus negatively influencing species number, this competition could be due to the absence of resources. In subprovinces with high levels of precipitation and climates low seasonal, competition is less pronounced allowing to maintain species with similar traits. The high environmental heterogeneity of Oaxaca allows exploration of the influence of different variables on the diversity of cricetids, through the ecological mechanisms of niche filtering.

Regarding the evaluation of relationships with environmental variables, we expected that the species richness was going to be explained by elevation, and our results partially supported this, as species richness is explained by the model that includes all of the environmental variables; however, a strong positive relationship was presented with elevation. For example, in the high elevation in the subprovince Sierra Madre de Oaxaca potentially recorded up to 36 species of cricetids, in contrast, on the coastal plains of the Gulf, the Pacific, and Tehuantepec, the cells recorded a maximum of 13 potentially coexisting species. This altitudinal pattern differs from that found in other studies of mice [33], although high values have been documented at high elevations in small mammals (marsupials and sigmodontine rodents) [100].

Unlike the pattern of species richness, high functional diversity (positive values of SES.FD indicating functional overdispersion) was highest in the subprovince Montañas y Valles del Centro and in the Sierra Madre del Sur de Oaxaca y Chiapas, at intermediate altitudes. For this reason, the environmental variables explain very little of the variance in the high functional diversity. On the contrary, low functional diversity (negative values of SES.FD that indicate functional clustering; [101]) was recorded in the lowlands of the Planicie Costera del Pacífico and the northern part of the Montañas y Valles del Occidente, at low elevations. As expected, the GLMs and GAMs results indicate that the functional diversity of cricetid mice could be explained by the interrelationships among environmental variables. Perhaps the AMT, AMP, PET and NPP produced environmental heterogeneity, allowing the formation of different habitats that could be explored and subsequently used by species with different functional traits. However, in these multiple models, environmental variables were not good predictors of the positive values of SES, probably due to the variation in these values.

Other studies conducted at regional scales have also found that environmental and habitat conditions are related to species richness and functional diversity [102–105]. For this reason, the mechanism that could be operating at this scale is that of environmental filtering, which implies that the species that coexist share more similarities than would be expected by chance, since the environmental conditions act as a filter, causing only certain traits to persist [106].

However, it is not only environmental factors, but also different historic and ecological processes [10, 98] that could shape the structure of the assemblages of the communities of cricetid rodents in Oaxaca, as stated in other studies [6, 97, 98]. On one hand, some historic processes could be related to the shared biogeographic origin of rodents [16, 17]. The geographic affinities could explain why the species inhabit certain altitudes, climates and vegetation types with affinity to those derived from their biogeographic origin [16]. On the other hand, different ecological mechanisms, such as ecological interactions [107] like interspecific competition of species with similar traits, could have an influence on the patterns of spatial distribution of biodiversity [108]. Thus, the distribution of species could be restricted not only by their physiological limits, but also by the stress of resources competition. Then, the niche conservatism would be an important evolutionary force driving patterns of the diversity of mammal’s assemblages [60].

## Conclusions

The spatial incongruence found between species richness and functional diversity of cricetid rodents indicates the great challenge of prioritizing biodiversity conservation in zones of elevated heterogeneity, such as Oaxaca. For this reason, the integration of knowledge about different dimensions of biodiversity will help conservation planning, e.g., for selection of protected areas. If this is based only on one dimension or on traditional metrics, such as species richness, the ecological roles of species that are key to the maintenance and function of the ecosystems could be masked. We suggest that assessing the protected areas in Oaxaca (of both social and governmental initiative) is crucial in order to determine whether these areas truly comply with conservation, since other studies have evidenced that functional diversity in protected natural areas is underrepresented compared to taxonomic diversity [109].

Several questions remain to be explored regarding the diversity of rodents in Oaxaca. For example, it would be very interesting to analyze the spatial patterns of beta diversity and phylogenetic diversity of the cricetids in order to determine the current geographic distribution of the evolutionary relationships among the species. In addition, basic ecological studies of various cricetid species are necessary in order to provide information about functional traits of importance to the species. It would also be interesting to contrast the patterns of different biological groups in Oaxaca, given its high environmental, climatic and physiographic variation, since it could be considered a natural laboratory for exploring the mechanisms that regulate the spatial distribution of biodiversity, a pattern that could be found in sites with similar characteristics.

## Supporting information

S1 Table Brief description of the physiographic subprovinces of Oaxaca, Mexico. (PDF)

S2 Table Parameters and results of potential species distribution model. (PDF)

S3 Table Evaluation of spatial congruence between measures of biodiversity. (PDF)

S4 Table Correlations among environmental variables. (PDF)

S5 Table Relationships between environmental variables and species richness and functional diversity of cricetids. (PDF)

S1 Text Correlograms and semivariograms of the Generalized Linear Model and Generalized Additive Models. (PDF)

S1 Fig Spatial congruence between species richness and observed functional diversity (FD). (PDF)
