# Supplementary material for: Spatial incongruence in the species richness and functional diversity of cricetid rodents
Source: PLoS One. 2019 Jun 7;14(6):e0217154. doi: 10.1371/journal.pone.0217154 (PMC6555520; doi:10.1371/journal.pone.0217154)
Supplement: S1 Table — (PDF) [file pone.0217154.s001.pdf]

Spatial incongruence in the species richness and functional diversity of cricetid rodents

Cintia Natalia Martín-Regalado, Miguel Briones-Salas, Mario C. Lavariega and Claudia E. Moreno

S1 Table. Brief description of the physiographic subprovinces of Oaxaca, Mexico, following Ortíz-Pérez et al. (2004).

Physiographic subprovinces	Characteristics
1. Depresión del Balsas (DB)	It is dominated by topography of low elevations, with an altitudinal interval that goes from 1200 to 1800 m a.s.l. The climate is warm and dry. The most characteristic vegetation types are tropical dry forest, scrub forest, and oak forest.
2. Montañas y Valles del Occidente (MVO)	It is a system of mountains with a generalized direction north-south, which form a cusp when reaching 3400 m a.s.l. It includes a climatic gradient, being warm-dry in the lowlands, dry semi-humid at mid elevations, and cold humid at high elevations. In the lowlands, there are dry forests, followed by oak forest in the intermediate, and pine forest in the highest parts.
3. Fosa de Tehuacán (FT)	Presents mostly elevations below 1000 m a.s.l., in which a semiarid climate dominates, with the predominance of tropical dry forest and various types of scrub, mainly xeromorphic.
4. Sierra Madre de Oaxaca (SMO)	It is a mountainous area with an average elevation above 2500 m a.s.l. The humid-temperate is the dominant climate, where there are fragments of cloud forests, *Quercus*, *Pinus*, and *Abies* forests; as well as small areas with scrubs in the lowlands.
5. Planicie Costera del Golfo (PCG)	It essentially has a plain topography, with a 90% of the region not exceeding the 400 m a.s.l. Climate is warm-humid. The evergreen rain forests are the main vegetation types; small areas are interspersed with savannas, oak, and tropical pine forests.
6. Valles Centrales de Oaxaca (VCO)	About 60% of the surface is below 1600 m a.s.l. The climate is warm-dry. The original vegetation, tropical dry forest, was eliminated for the establishment of agriculture, but in the foothills there are fragments of *Quercus* and *Pinus* forests.
7. Montañas y Valles del Centro (MVC)	It is an area with mountains and valleys, where the most notable mountain reaches the 2800 m a.s.l. This subprovince presents warm-dry and temperate climates.

	The predominant vegetation types are *Quercus-Pinus* forest, cloud forest, and evergreen forest; in some areas, there are scrubs and tropical dry forest.
8. Depresión del Istmo de Tehuantepec (DIT)	It is a relatively depressed area; the main montane range reaches 400 m a.s.l. The climate is warm-dry, allowing the presence of thorny tropical dry forest, savannas, scrubs, and small areas with *Quercus* and *Pinus* forests.
9. Sierra Madre del Sur de Oaxaca y Chiapas (SMSOC)	More than 20% of the surface is above 1000 m a.s.l. In the low areas, the climate is warm-humid that changes to temperate humid in the high zones. Presents evergreen forests and cloud forests.
10. Sierra Madre del Sur (SMS)	The relief is contrasting but essentially the mountains have an altitude of 2000 m. In this subprovince, the dominant climate is temperate humid. The main vegetation types are *Quercus* and *Pinus* forest, cloud forest, and evergreen forest; in very restricted areas there are scrubs and tropical dry forests.
11. Planicie Costera del Pacífico (PCP)	It can be divided into two areas, one with a soft relief, where plains are the main characteristic, and another with a premontane relief in mid-elevations. The climate in the plains is warm-dry. The dry forests are the main vegetation type, however, in the ravines and most humid areas a sub perennial type can be found; on the coastline, there are fragments of mangroves.
12. Planicie Costera de Tehuantepec (PCT)	It is the region with the plainest topography of all the subprovinces, with 90% being plain and below 200 m a.s.l. The climate is warm-dry, where savannas, scrubs, and mangroves prevail. In the coastal lagoons, there are mangroves.

Ortiz-Pérez MA, Hernández-Santana JR, Figueroa-Mah-Eng JM. Reconocimiento fisiográfico y geomorfológico. In: García-Mendoza AJ, Ordóñez MJ, Briones-Salas M, editors. Biodiversidad de Oaxaca. México, D.F. Instituto de Biología, Universidad Nacional Autónoma de México-Fondo Oaxaqueño para la Conservación de la Naturaleza-World Wildlife Found; 2004. pp. 43-54.
