# Supplementary material for: Spatial incongruence in the species richness and functional diversity of cricetid rodents
Source: PLoS One. 2019 Jun 7;14(6):e0217154. doi: 10.1371/journal.pone.0217154 (PMC6555520; doi:10.1371/journal.pone.0217154)
Supplement: S2 Table — (PDF) [file pone.0217154.s002.pdf]

Spatial incongruence in the species richness and functional diversity of cricetid rodents

Cintia Natalia Martín-Regalado, Miguel Briones-Salas, Mario C. Lavariega and Claudia E. Moreno

S2 Table. Parameters and results of potential species distribution models of cricetid rodents. For *Microtus umbrosus* we did not perform ecological niche modeling because the records were restricted to a single locality, and therefore its distribution was considered as a single cell.

Species	Presence records	Percentage to training	Variables used	AUC	Threshold
Microtus mexicanus	98	60	alt, bio1, bio2, bio3, bio5, bio7, bio8, bio9, bio10, bio12, bio14, bio15, bio18, bio19	0.95	10 percentile training presence (0.063)
Microtus oaxacensis	11	82	alt, bio7, bio8, bio11, bio14, bio17, bio18, bio19	0.98	Minimum training presence (0.446)
Microtus quasiater	18	83	alt, bio2, bio2, bio3, bio4, bio6, bio7, bio12, bio13, bio14, bio15, bio16, bio19	0.99	Minimum training presence (0.024)
Baiomys musculus	86	58	alt, bio3, bio4, bio5,bio6, bio7,bio11,bio13,bio14,bio15, bio18, bio19	0.90	10 percentile training presence (0.302)
Scotinomys teguina	29	100	alt, bio2, bio3, bio4, bio5, bio6, bio7, bio8, bio19, bio12, bio14, bio18	0.90	Minimum training presence (0.027)
Hodomys alleni	31	100	alt, bio3, bio4, bio8, bio11, bio12, bio13, bio14, bio15, bio16, bio18, bio19	0.93	Minimum training presence (0.118)
Neotoma mexicana	126	60	alt, bio1, bio2, bio3, bio4, bio5, bio8, bio9, bio12, bio14, bio15, bio18, bio19	0.90	10 percentile training presence (0.278)
Habromys chinanteco	5	100	alt, bio2, bio4, bio7, bio12, bio15, bio18,	0.92	Minimum training

			bio19		presence (0.468)
Habromys ixtlani	17	82	alt, bio2, bio3, bio6, bio7, bio9, bio11, bio14, bio16, bio19	0.99	Minimum training presence (0.218)
Habromys lepturus	5	100	alt, bio1, bio2, bio3, bio5, bio6, bio8, bio9, bio12, bio18, bio19	0.96	Minimum training presence (0.247)
Habromys simulatus	7	100	alt, bio2, bio3, bio4, bio5, bio9, bio10, bio11, bio13, bio14, bio15	0.94	Minimum training presence (0.495)
Megadontomys cryophilus	20	75	alt, bio2, bio3, bio7, bio12, bio15, bio18	0.96	Minimum training presence (0.504)
Megadontomys nelsoni	10	100	alt, bio2, bio3, bio4, bio8, bio11, bio15, bio16, bio17, bio18, bio19	0.98	Minimum training presence (0.440)
Megadontomys thomasi	54	69	alt, bio2, bio3,bio4, bio6, bio7, bio12, bio13, bio14, bio15, bio18, bio19	0.95	Minimum training presence (0.013)
Peromyscus aztecus	211	60	alt, bio4, bio5, bio6, bio7, bio12, bio15, bi015	0.93	10 percentile training presence (0.146)
Peromyscus beatae	78	71	alt, bio1, bio2, bio4, bio5, bio9, bio11, bio14, bio15, bio16, bio19	0.98	10 percentile training presence (0.125)
Peromyscus difficilis	94	78	alt, bio1, bio2, bio4, bio5, bio9, bio12, bio14, bio15, bio18, bio19	0.93	10 percentile training presence (0.145)
Peromyscus furvus	155	57	alt, bio1, bio2, bio3, bio5, bio6, bio9, bio10, bio12, bio13, bio14, bio15, bio19	0.98	10 percentile training presence (0.360)
Peromyscus gratus	189	70	alt, bio1, bio2, bio3, bio9, bio11, bio15, bio16, bio17, bio18, bio19	0.90	10 percentile training presence (0.344)

Peromyscus leucopus	56	72	alt, bio3, bio4, bio6, bio7, bio12, bio13, bio14,bio15, bio16	0.92	Minimum training presence (0.158)
Peromyscus maniculatus	130	60	alt, bio2, bio3, bio4, bio6, bio8, bio10, bio13, bio14, bio15, bio18, bio19	0.94	10 percentile training presence (0.137)
Peromyscus megalops	76	71	alt, bio4, bio6, bio7, bio10, bio15, bio16, bio17, bio18, bio19	0.96	10 percentile training presence (0.152)
Peromyscus melanocarpus	54	70	alt, bio5, bio6, bio7, bio15, bio19	0.93	Minimum training presence (0.130)
Peromyscus melanophrys	104	79	alt, bio2, bio4, bio9, bio11, bio13, bio15, bio17, bio18, bio19	0.89	10 percentile training presence (0.376)
Peromyscus melanotis	61	59	alt, bio1, bio2, bio4, bio5, bio9, bio10, bio15, bio17, bio19	0.98	10 percentile training presence (0.070)
Peromyscus melanurus	17	82	alt, bio2, bio3, bio4, bio15, bio18, bio19	0.91	Minimum training presence (0.254)
Peromyscus mexicanus	124	89	alt, bio2, bio4, bio9, bio11, bio13, bio15, bio17, bio18, bio19	0.90	10 percentile training presence (0.370)
Reithrodontomys fulvescens	204	61	alt, bio1, bio2, bio3, bio4, bio5, bio8, bio9, bio12, bio13, bio14, bio15, bio18, bio19	0.95	10 percentile training presence (0.335)
Reithrodontomys megalotis	67	70	alt, bio1, bio2, bio3, bio4, bio5, bio7, bio8, bio9, bio10, bio12, bio13, bio14, bio15, bio18, bio19	0.95	10 percentile training presence (0.174)
Reithrodontomys mexicanus	100	74	alt, bio2, bio3, bio4, bio7, bio11, bio13, bio15, bio17, bio18, bio19	0.90	10 percentile training presence (0.184)
Reithrodontomys	35	80	alt, bio12, bio14,	0.91	Minimum

microdon			bio15, bio17, bio18		training presence (0.057)
Reithrodontomys sumichrasti	42	55	alt, bio2, bio3, bio4, bio5, bio12, bio15, bio17, bio18, bio19	0.92	Minimum training presence (0.195)
Oligoryzomys fulvescens	26	70	alt, bio2, bio3, bio7, bio11, bio12, bio13, bio15, bio17, bio18, bio19	0.80	Minimum training presence (0.118)
Oryzomys alfaroi	129	79	alt, bio1, bio5, bio6, bio7, bio9, bio19, bio14, bio15, bio16, bio19	0.92	10 percentile training presence (0.147)
Oryzomys chapmani	65	71	alt, bio2, bio3, bio4, bio5, bio6, bio12, bio14, bio15, bio18, bio19	0.92	10 percentile training presence (0.156)
Oryzomys couesi	203	90	alt, bio2, bio3, bio4, bio6, bio7, bio9, bio13, bio14, bio15, bio16, bio19	0.90	10 percentile training presence (0.281)
Oryzomys guerrerensis	21	100	alt, bio1, bio2, bio3, bio4, bio5, bio6, bio12, bio14, bio15, bio18, bio19	0.93	Minimum training presence (0.219)
Oryzomys melanotis	56	75	alt, bio3, bio4, bio5, bio7, bio15, bio16, bio17, bio18, bio19	0.94	Minimum training presence (0.116)
Oryzomys fulgens	16	100	alt, bio2, bio3, bio4, bio5, bio6, bio7, bio8, bio10, bio11, bio12, bio13, bio14, bio15, bio17, bio18, bio19	0.89	Minimum training presence (0.048)
Oryzomys rostratus	42	67	alt, bio2, bio6, bio7, bio9, bio12, bio14, bio15, bio18, bio19	0.91	Minimum training presence (0.060)
Rheomys mexicanus	3	100	alt, bio2, bio3, bio5, bio7, bio9, bio13	0.94	Minimum training presence (0.562)
Sigmodon alleni	47	70	alt, bio2, bio4, bio5, bio6, bio11, bio12,	0.91	Minimum training

			bio14, bio15, bio18, bio19		presence (0.229)
Sigmodon leucotis	44	64	alt, bio1, bio2, bio4, bio5, bio6, bio9, bio13, bio15, bio18, bio19	0.93	Minimum training presence (0.229)
Sigmodon mascotensis	146	78	alt, bio1, bio1, bio4, bio5, bio9, bio13, bio15, bio17, bio18, bio19	0.94	10 percentile training presence (0.330)
Sigmodon planifrons	3	100	alt, bio2, bio4, bio14, bio19	0.90	Minimum training presence (0.450)
Sigmodon toltecus	127	78	alt, bio2, bio3, bio4, bio6, bio7, bio15, bio16, bio17, bio18, bio19	0.83	10 percentile training presence (0.326)
Nyctomys sumichrasti	83	78	alt, bio2, bio4, bio5, bio7, bio9, bio12, bio13, bio15, bio16, bio17, bio18, bio19	0.78	10 percentile training presence (0.238)
Tylomys nudicaudus	55	77	alt, bio2, bio3, bio4, bio6, bio7, bio9, bio12, bio13, bio16, bio17, bio18, bio19	0.89	Minimum training presence (0.253)

Abbreviations: alt=altitude, bio1=annual mean temperature, bio2=mean diurnal range (mean of monthly (max temp-min temp)), bio3=isothermality (bio2/bio7)(*100), bio4=temperature seasonality (standard deviation*100), bio5=max temperature of warmest month, bio6= min temperature of coldest month, bio7=temperature annual range (bio5-bio6), bio8=mean temperature of wettest quarter, bio9=mean temperature of driest quarter, bio10=mean temperature of warmest quarter, bio11=mean temperature of coldest quarter, bio12= annual precipitation, bio13=precipitation of wettest month, bio14=precipitation of driest month, bio15=precipitation seasonality (coefficient of variation), bio16=precipitation of wettest quarter, bio17= precipitation of driest quarter, bio18=precipitation of warmest quarter, bio19=precipitation of coldest quarter. AUC: Area Under the Curve.
