# Supplementary material for: Spatial incongruence in the species richness and functional diversity of cricetid rodents
Source: PLoS One. 2019 Jun 7;14(6):e0217154. doi: 10.1371/journal.pone.0217154 (PMC6555520; doi:10.1371/journal.pone.0217154)
Supplement: S3 Table — (PDF) [file pone.0217154.s003.pdf]

Spatial incongruence in the species richness and functional diversity of cricetid rodents

Cintia Natalia Martín-Regalado, Miguel Briones-Salas, Mario C. Lavariega and Claudia E. Moreno

S3 Table. Evaluation of spatial congruence between measures of biodiversity. The table includes percentages of communities per category of congruence, for species richness and functional diversity without the richness effect (SES.FD), at five resolutions. The colors match with the Figure 2 of the manuscript: green=high congruence, light orange=moderate congruence, and orange=incongruence.

Categories	Low richness (3-12 species)	Moderate richness (13-22 species)	High richness (23-32 species)	Total
**Resolution: 25 km^2**				
SES.FD low (-3.77 - -1.52)	0.20	4.97	4.11	9.28
SES.FD moderate (-1.51 - 0.73)	3.18	30.48	32.06	65.72
SES.FD high (0.72 - 2.98)	2.09	15.73	7.18	25.00
Total	5.47	51.18	43.35	100
**Resolution: 50 km^2**				
SES.FD low (-3.61 - -1.65)	0.20	3.83	3.09	7.12
SES.FD moderate (-1.64 - 0.30)	6.77	20.47	22.29	49.53
SES.FD high (0.29 - 2.26)	12.08	16.69	14.58	43.35
Total	19.05	40.99	39.96	100
**Resolution: 100 km^2 with the functional diversity (FD) index**				
FD low (3.21 - 4.60)	22.95	0	0	22.95
FD moderate (4.61 - 6.00)	4.07	26.99	0	31.06
FD high (6.01 - 7.4)	0	12.02	33.97	45.99
Total	27.02	39.01	33.97	100

Categories	Low richness (3-12 species)	Moderate richness (13-22 species)	High richness (23-32 species)	Total
**Resolution: 100 km^2 with standardized effect size of functional diversity (SES.FD)**				
SES.FD low (-3.06 - -1.31)	1.74	8.42	2.66	12.82
SES.FD moderate (-1.30 - 0.42)	9.34	17.77	20.79	47.90
SES.FD high (0.43 - 2.17)	15.66	13.00	10.62	39.28
Total	26.74	39.19	34.07	100
**Resolution: 200 km^2**				
SES.FD low (-2.74 - -1.15)	0.73	5.84	8.58	15.15
SES.FD moderate (-1.14 - 0.45)	5.29	14.78	30.11	50.18
SES.FD high (0.44 - 2.04)	7.66	14.23	12.78	34.67
Total	13.68	34.85	51.47	100
**Resolution: 400 km^2**				
SES.FD low (-2.79 - -1.79)	0	5.32	0.36	5.68
SES.FD moderate (-1.78 - 0.26)	12.06	23.40	19.86	55.32
SES.FD high (0.25 - 1.79)	14.18	15.25	9.57	39.00
Total	26.24	43.97	29.79	100
