# Supplementary material for: Spatial incongruence in the species richness and functional diversity of cricetid rodents
Source: PLoS One. 2019 Jun 7;14(6):e0217154. doi: 10.1371/journal.pone.0217154 (PMC6555520; doi:10.1371/journal.pone.0217154)
Supplement: S4 Table — (PDF) [file pone.0217154.s004.pdf]

Spatial incongruence in the species richness and functional diversity of cricetid rodents

Cintia Natalia Martín-Regalado, Miguel Briones-Salas, Mario C. Lavariega and Claudia E. Moreno

S4 Table. Correlations among environmental variables. Pairwise correlations among all variables (Pearson coefficient). In all cases P value is lower than 0.001. Elevation: mean elevation value per cell; AMT: Annual mean temperature value averaged per cell; AMP: Annual mean precipitation value averaged per cell; NPP: Net primary productivity; PET: mean potential evapotranspiration per cell.

	**AMT**	**AMP**	**NPP**	**PET**
**Elevation**	-0.9717	-0.3932	-0.3827	-0.4928
**AMT**		0.2573	0.4004	0.6562
**AMP**			0.1179	-0.1817
**NPP**				0.1857
