# Supplementary material for: Spatial incongruence in the species richness and functional diversity of cricetid rodents
Source: PLoS One. 2019 Jun 7;14(6):e0217154. doi: 10.1371/journal.pone.0217154 (PMC6555520; doi:10.1371/journal.pone.0217154)
Supplement: S5 Table — (PDF) [file pone.0217154.s005.pdf]

Spatial incongruence in the species richness and functional diversity of cricetid rodents

Cintia Natalia Martín-Regalado, Miguel Briones-Salas, Mario C. Lavariega and Claudia E. Moreno

S5 Table. Relationships between environmental variables and species richness and functional diversity of cricetids. The table includes the results of the univariate and multivariate Generalized Lineal Models (GLMs) and Generalized Additive Models (GAMs) for species richness and functional diversity SES positive and negative values (response variables). For the GAMs the variables were smoothed (marked with "s"). The description of the variables is in Table 2. Standard Error (SE), *** p<0.001, Akaike Information Criterion (AIC).

Response variable	Type	Model	Coefficients	Estimate	SE	t	F	P	R^2 adjusted	AIC
**Species richness**	Univariate models	GLM	Elevation	0.0002	0.0000	37.8		***	0.57	6241
		GAM	sElevation				437.2	***	0.77	5831
		GLM	AMT	-0.0051	0.0001	-39.3		***	0.58	6205
		GAM	sAMT				312.3		0.73	5937
		GLM	AMP	-0.0000	0.0000	-12.0		***	0.02	4556
		GAM	sAMP				7.74	***	0.03	4633
		GLM	NPP	-0.0000	0.0000	-13.8		***	0.17	5046
		GAM	sNPP				32.63	***	0.22	5279
		GLM	PET	-0.0006	0.0000	-14.7		***	0.15	5100
		GAM	sPET				45.45	***	0.25	6855
	Multivariate model: AMT+AMP+ NPP+PET	GLM							0.64	6102
			AMT	-0.0066	0.0002	-31.7		***		
			AMP	0.0000	0.0000	9.0		***		
			NPP	-0.0000	0.0000	-5.1		***		
			PET	0.0004	0.0000	11.0		***		
		GAM							0.78	5844
			sAMT				212.74	***		
			sAMP				10.96	***		
			sNPP				8.91	***		
			sPET				20.31	***		
**SES.FD positive values**	Univariate models	GLM	Elevation	-0.0000	0.0000	-1.8		0.6	0.005	676
		GAM	sElevation				2.10	***	0.011	671
		GLM	AMT	0.0003	0.0004	0.8		0.5	0.002	678
		GAM	sAMT				2.42	0.07	0.010	684
		GLM	AMP	-0.0000	0.0000	-3.8		***	0.002	663
		GAM	sAMP				9.82	***	0.109	608
		GLM	NPP	0.0000	0.0000	1.7		0.08	0.005	676
		GAM	sNPP				8.8	***	0.100	616
		GLM	PET	-0.0004	0.0001	-4.4		***	0.030	660
		GAM	sPET				6.4	***	0.039	653
	Multivariate model:	GLM							0.18	567
			AMT	0.0047	0.0005	8.1		***		

Response variable	Type	Model	Coefficients	Estimate	SE	t	F	P	R^2 adjusted	AIC
	AMT+AMP +NPP+PET		AMP	-0.0002	0.0000	-8.9		***		
			NPP	0.0000	0.0000	0.4		0.6		
			PET	-0.0014	0.0001	-10.2		***		
		GAM							0.27	493
			sAMT				12.7	**		
			sAMP				14.1	***		
			sNPP				2.03	0.08		
			sPET				18.6	***		
**SES.FD negative values**	Univarate models	GLM	Elevation	-0.0001	0.0000	-4.4		***	0.043	995
		GAM	sElevation				7.15	***	0.085	978
		GLM	AMT	0.0023	0.0008	2.8		**	0.018	1009
		GAM	sAMT				4.8	***	0.075	985
		GLM	AMP	0.0005	0.0000	11.1		***	0.220	905
		GAM	sAMP				28.0	***	0.350	827
		GLM	NPP	0.0000	0.0000	4.8		***	0.049	994
		GAM	sNPP				10.2	***	0.106	967
		GLM	PET	-0.0007	0.0001	-4.0		***	0.036	1001
		GAM	sPET				5.4	***	0.049	994
	Multivariate model: AMT+AMP +NPP+PET	GLM							0.31	854
			AMT	0.0076	0.0014	5.4		***		
			AMP	0.0003	0.0000	6.7		***		
			NPP	0.0000	0.0000	1.4		0.15		
			PET	-0.0019	0.0002	-7.2		***		
		GAM							0.46	759
			sAMT				3.31	***		
			sAMP				13.4	***		
			sNPP				1.10	0.35		
			sPET				7.35	***		
