# Supplementary material for: Spatial incongruence in the species richness and functional diversity of cricetid rodents
Source: PLoS One. 2019 Jun 7;14(6):e0217154. doi: 10.1371/journal.pone.0217154 (PMC6555520; doi:10.1371/journal.pone.0217154)

Spatial incongruence in the species richness and functional diversity of cricetid rodents

Cintia Natalia Martín-Regalado, Miguel Briones-Salas, Mario C. Lavariega and Claudia E. Moreno

S1 Text. Correlograms and semivariograms of the generalized linear models and generalized additive models.

In this document we include the correlograms showing the autocorrelation in species richness, SES.FD positive and negative values, see the Figure below. Moran's I statistics show that variables were strongly autocorrelated (p~0). Thus, to compensate this autocorrelation, we included coordinates into models, as we explain in the manuscript. To demonstrate the effectivity in controlling spatial autocorrelation with the mentioned approach, the figures on the Table below compare semivariograms of residuals, showing that the residuals are less spatially correlated in GAMs than GLMs.

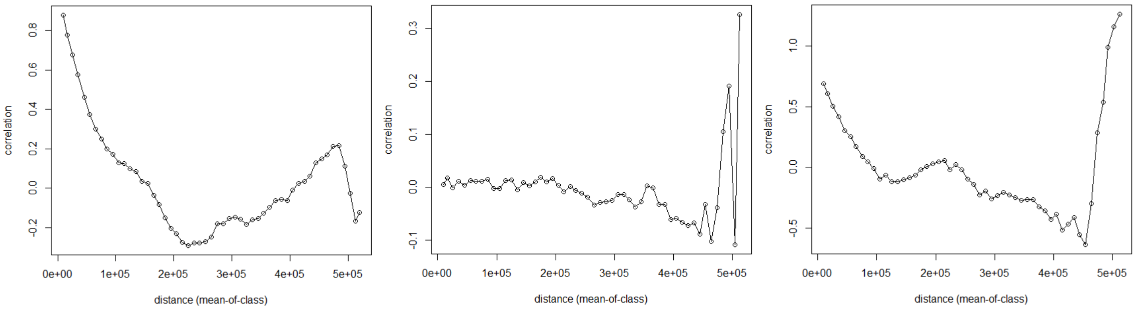

Figure with correlograms of spatial autocorrelation in response variable. Left, Species richness; center, SES.FD positive values; right, SES.FD negative values.

Table with the semivariograms of residuals on GLMs and GAMs models. The description of the variables is in Table 2 in the manuscript.

<table>
<tr><th>Generalized Linear Models (GLMs)</th><th>Generalized Additive Models (GAMs)</th></tr>
<tr><td colspan="2">Species Richness~Elevation+AMT+AMP+NPP+PET</td></tr>
<tr><td>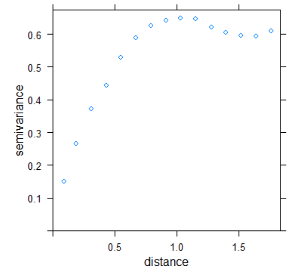
</td><td>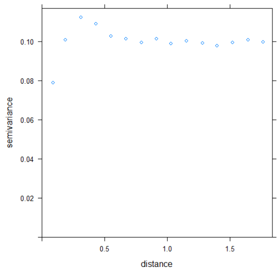
</td></tr>
<tr><td colspan="2">Species Richness~Elevation+AMP+NPP+PET</td></tr>
<tr><td>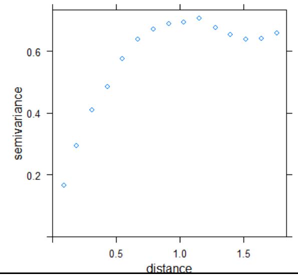
</td><td>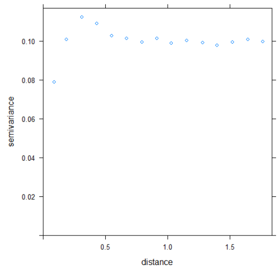
</td></tr>
<tr><td colspan="2">Species Richness~AMT +AMP+NPP+PET</td></tr>
<tr><td>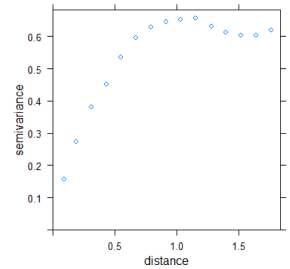
</td><td>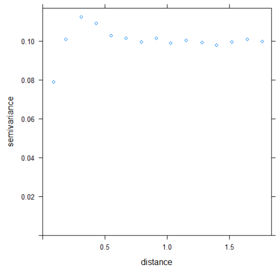
</td></tr>
<tr><td colspan="2">Species Richness~Elevation</td></tr>
<tr><td>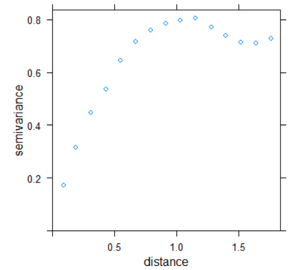
</td><td>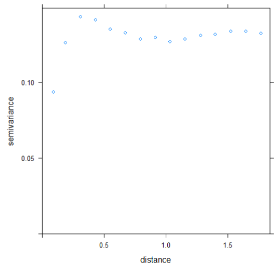
</td></tr>
<tr><td colspan="2">Species Richness~AMT</td></tr>
<tr><td>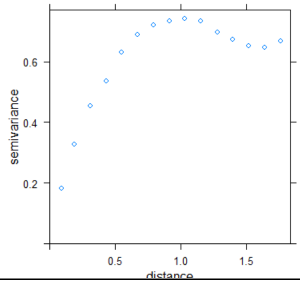
</td><td>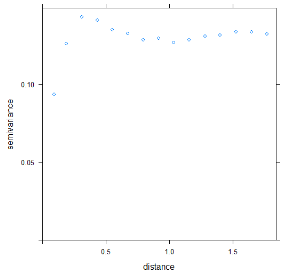
</td></tr>
</table>

Species Richness~AMP

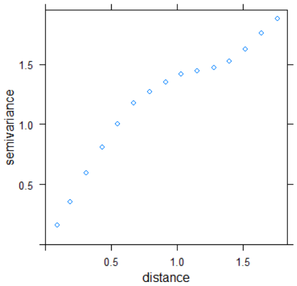

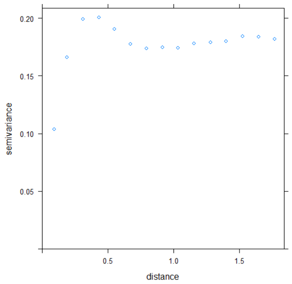

Species Richness~NPP

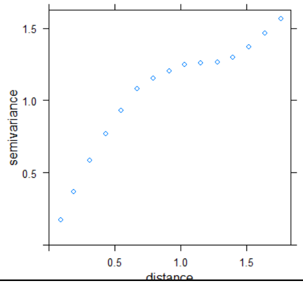

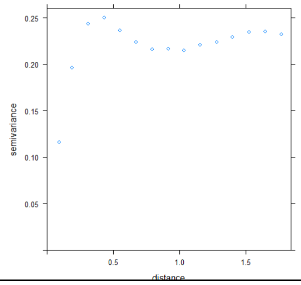

Species Richness~PET

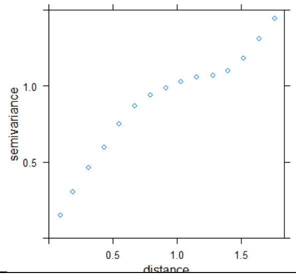

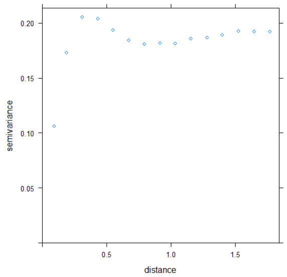

SES.FD positive~Elevation+AMT+AMP+NPP+PET

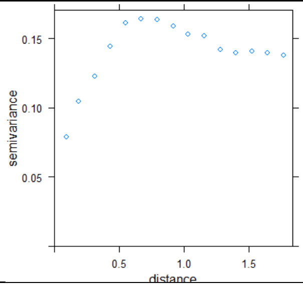

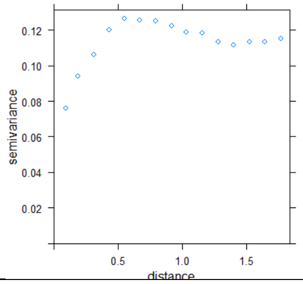

SES.FD positive~Elevation+AMP+NPP+PET

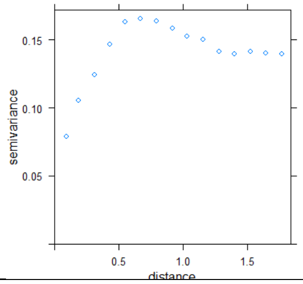

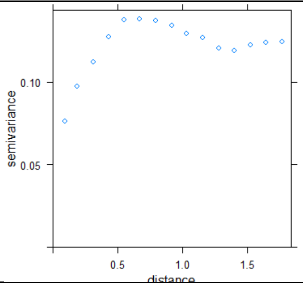

SES.FD positive~AMT+AMP+NPP+PET

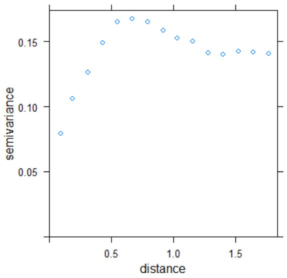

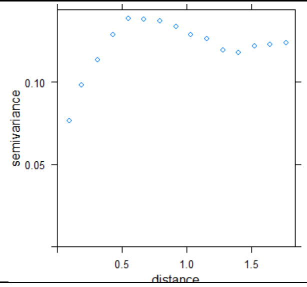

SES.FD positive~Elevation+AMP+PET

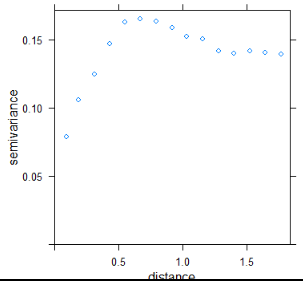

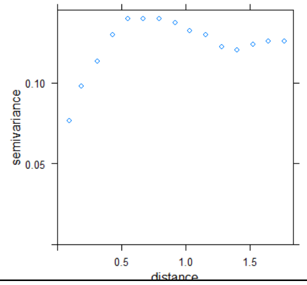

SES.FD positive~AMT+AMP+PET

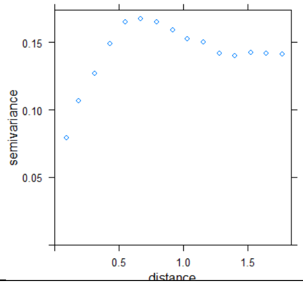

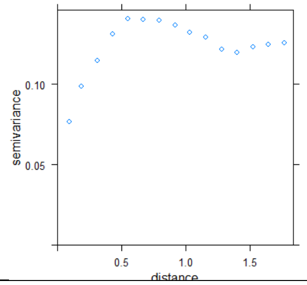

SES.FD positive~Elevation

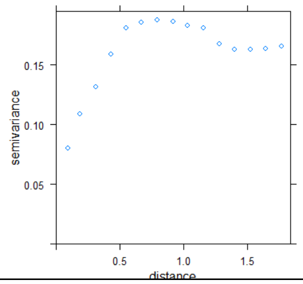

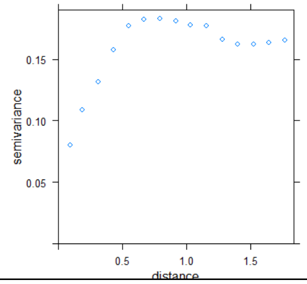

SES.FD positive~AMT

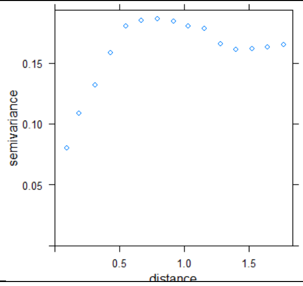

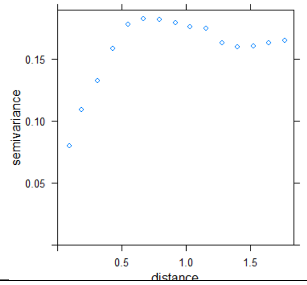

SES.FD positive~AMP

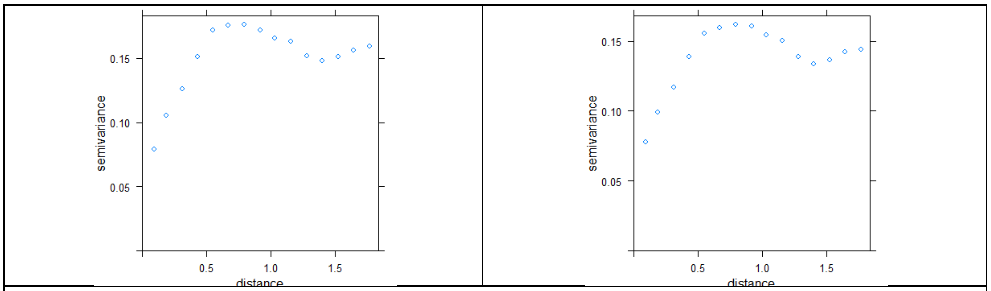

SES.FD positive~NPP

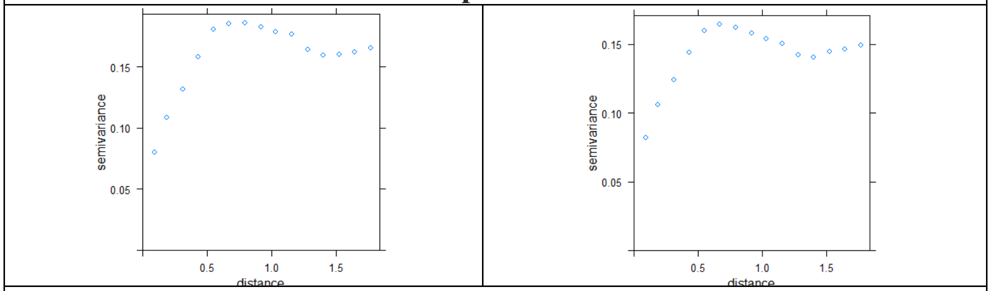

SES.FD positive~PET

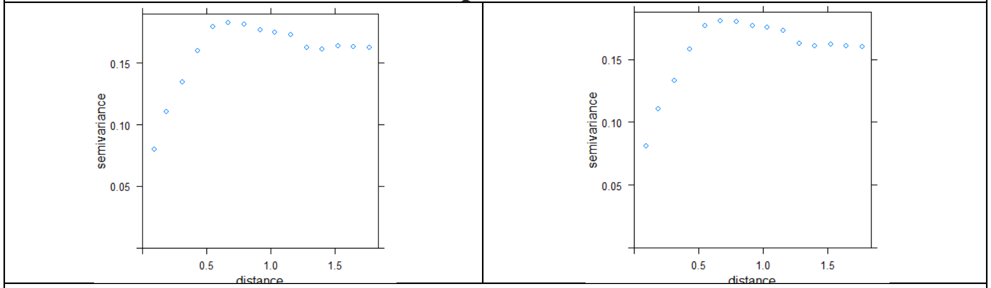

SES.FD negative~Elevation+AMT+AMP+NPP+PET

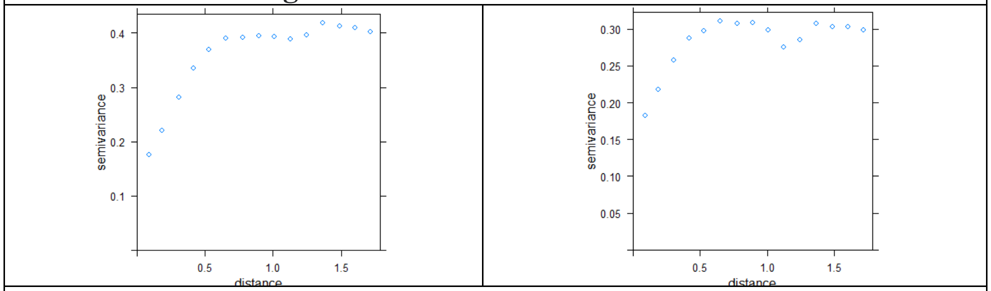

SES.FD negative~Elevation+AMP+NPP+PET

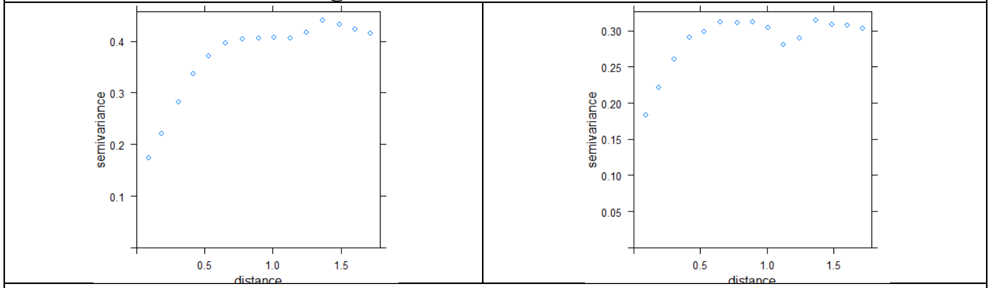

SES.FD negative~Elevation+AMP+AMP+PET

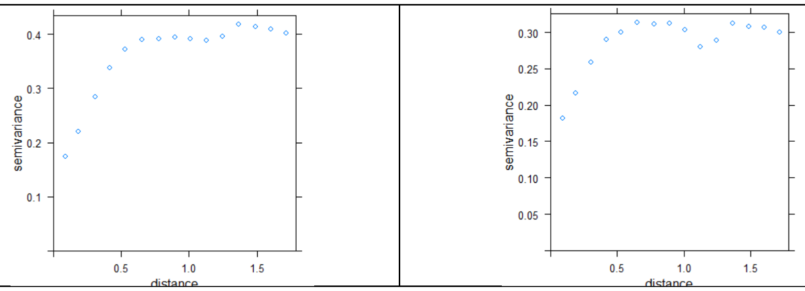

SES.FD negative~Elevation+AMP+PET

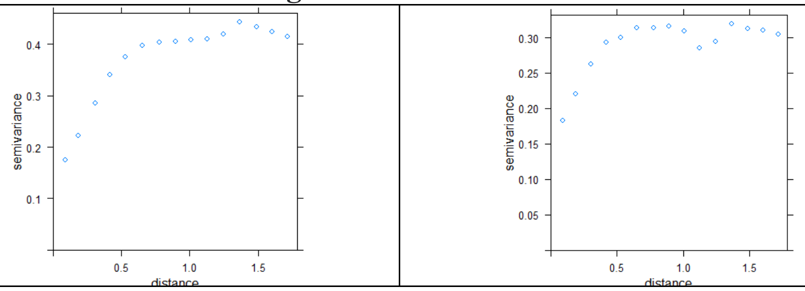

SES.FD negative~AMT+AMP+NPP+PET

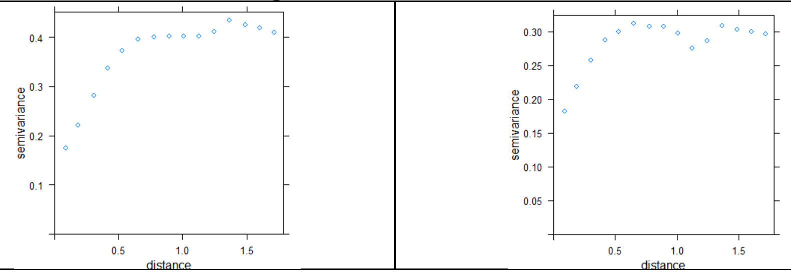

SES.FD negative~Elevation

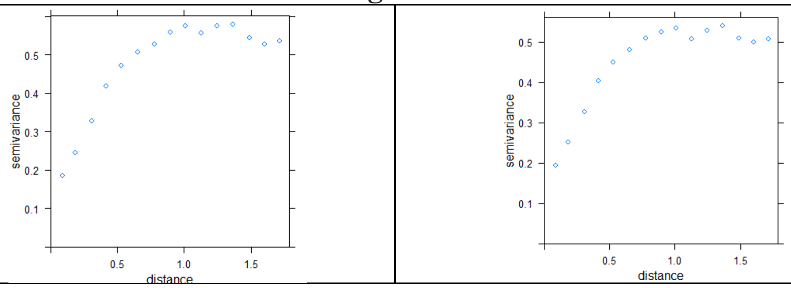

SES.FD negative~AMT

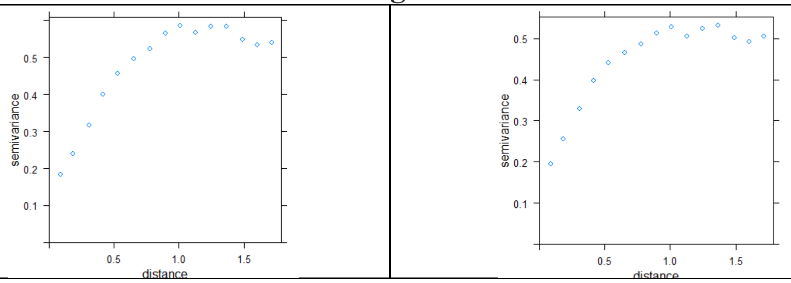

SES.FD negative~AMP

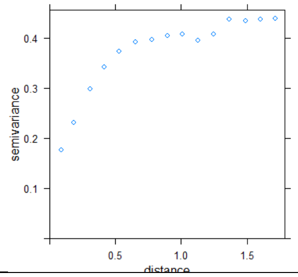

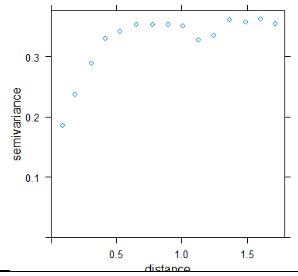

SES.FD negative~NPP

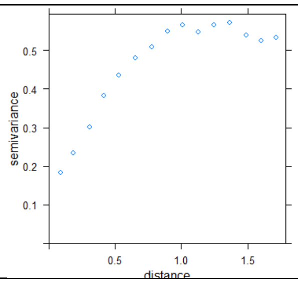

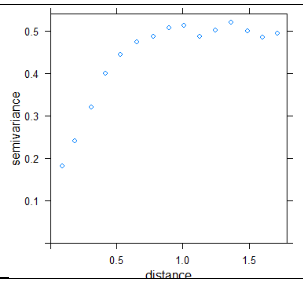

SES.FD negative~PET

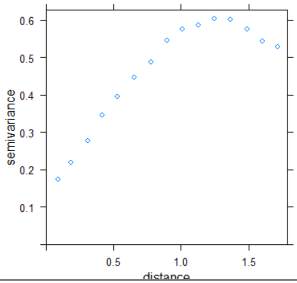

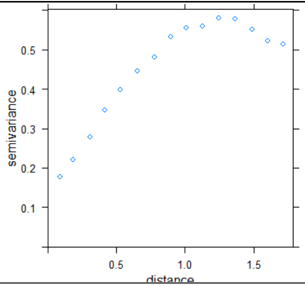

Supplement: S1 Text — (PDF) [file pone.0217154.s006.pdf]
