# Supplementary material for: Spatial incongruence in the species richness and functional diversity of cricetid rodents
Source: PLoS One. 2019 Jun 7;14(6):e0217154. doi: 10.1371/journal.pone.0217154 (PMC6555520; doi:10.1371/journal.pone.0217154)

Spatial incongruence in the species richness and functional diversity of cricetid rodents

Cintia Natalia Martín-Regalado, Miguel Briones-Salas, Mario C. Lavariega and Claudia E. Moreno

S1 Fig. Spatial congruence between species richness and observed functional diversity (FD). Physiographic subprovinces: Depresión del Balsas (DB), Montañas y Valles del Occidente (MVO), Fosa de Tehuacán (FT), Sierra Madre de Oaxaca (SMO), Planicie Costera del Golfo (PCG), Valles Centrales de Oaxaca (VCO), Montañas y Valles del Centro (MVC), Depresión del Istmo de Tehuantepec (DIT), Sierra Madre del Sur de Oaxaca y Chiapas (SMSOC), Sierra Madre del Sur (SMS), Planicie Costera del Pacífico (PCP) and Planicie Costera de Tehuantepec (PCT).

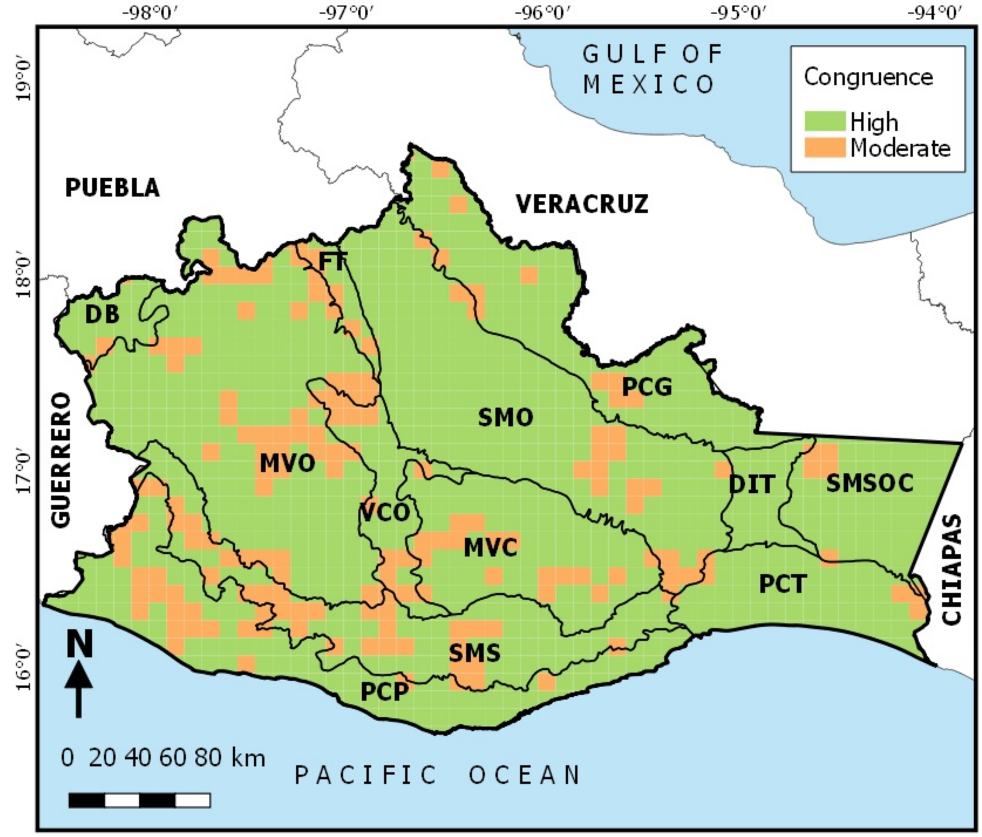

Supplement: S1 Fig — (PDF) [file pone.0217154.s007.pdf]
